# Outcomes of coronary revascularization in patients with metabolic dysfunction-associated steatotic liver disease: a systematic review

**DOI:** 10.3389/fcvm.2025.1609071

**Published:** 2025-08-26

**Authors:** Jacob J. Gries, Hafeez Ul Hassan Virk, Zhen Wang, Mahboob Alam, Samin Sharma, Markus Strauss, Chayakrit Krittanawong

**Affiliations:** ^1^ Department of Gastroenterology, Allegheny Health Network, Pittsburgh, PA, United States; ^2^ Harrington Heart & Vascular Institute, Case Western Reserve University, University Hospitals Cleveland Medical Center, Cleveland, OH, United States; ^3^ Robert D. and Patricia E. Kern Center for the Science of Health Care Delivery, Mayo Clinic, Rochester, MN, United States; ^4^ Division of Health Care Policy and Research, Department of Health Sciences Research, Mayo Clinic, Rochester, MN, United States; ^5^ The Texas Heart Institute, Baylor College of Medicine, Houston, TX, United States; ^6^ Cardiac Catheterization Laboratory of the Cardiovascular Institute, Mount Sinai Hospital, New York, NY, United States; ^7^ Department of Cardiology, Sector Preventive Medicine, Health Promotion, Faculty of Health, School of Medicine, University Witten/Herdecke, Hagen, Germany; ^8^ Department of Cardiology I—Coronary and Periphal Vascular Disease, Heart Failure Medicine, University Hospital Muenster, Muenster, Germany; ^9^ HumanX, Delaware, DE, United States

**Keywords:** coronary heart disease, MASLD, coronary revascularisation, liver disease, coronary disease, metabolic dysfunction-associated fatty liver disease (MASLD)

## Abstract

**Introduction:**

A growing amount of evidence suggests that metabolic dysfunction-associated steatotic liver disease (MASLD) may independently increase the risk of coronary artery disease and acute coronary syndrome, thus necessitating revascularization interventions such as percutaneous coronary intervention (PCI) and coronary artery bypass grafting (CABG) [2,3]. However, a limited number of studies have evaluated the impact of MASLD on the outcomes of these interventions.

**Methods:**

A comprehensive search of the PubMed/MEDLINE and Embase databases was conducted to identify relevant studies from August 2015 to August 2025 using a combination of Medical Subject Headings (MeSH) terms and text words related to MASLD and cardiovascular revascularization.

**Results:**

Two hundred nineteen papers from the PubMed/MEDLINE and Embase databases were reviewed. Six met the inclusion criteria ( Figure 1). Five studies covered PCI, and one covered CABG. Supplemental information was added using targeted PubMed/MEDLINE searches.

**Conclusions:**

MASLD may pose an increased risk of in-hospital and long-term mortality following PCI. Risks for cardiogenic shock, cardiac arrest, in-stent thrombosis, gastrointestinal bleeding, or invasive mechanical ventilation following PCI may also be increased. Further studies are needed to determine the optimal coronary revascularization method and post-revascularization medical therapy for patients with MASLD.

## Introduction

Metabolic dysfunction-associated steatotic liver disease (MASLD), formerly known as non-alcoholic fatty liver disease (FLD), is characterized by abnormal lipid accumulation in the liver. This can lead to increased hepatic inflammation, now known as metabolic dysfunction-associated steatohepatitis (MASH), and advanced fibrosis or cirrhosis (1). It has become a significant global health concern due to its increasing prevalence and strong link to cardiovascular disease (2). The disease has been linked to a 2–3 times higher risk of coronary artery disease (CAD) and a 35% increased risk of cardiovascular disease mortality, regardless of other common risk factors such as hypertension, dyslipidemia, type 2 diabetes, and gender (2, 3). Mendelian randomization studies confirm a significant causal relationship even after excluding genes associated with impaired VLDL secretion (2, 4). Patients with MASLD show higher rates of subclinical coronary atherosclerosis, as indicated by radiographic coronary artery calcium scores (2, 5). The severity of CAD correlates directly with MASLD severity, with more extensive CAD seen in advanced MASLD, possibly due to shared pathogenic processes causing hepatic and myocardial fibrosis (2, 6, 7). MASLD is also independently linked to double the rate of adverse cardiovascular events, including acute coronary syndrome and ischemic stroke, and related mortality (2, 8–13). A meta-analysis of 36 cohort studies involving over 5.8 million middle-aged individuals found that MASLD increases the long-term risk of cardiovascular events, with a pooled hazard ratio (HR) of 1.45 (95% CI 1.31–1.61), independent of age, sex, adiposity, type 2 diabetes, and other risk factors. This risk is higher with advanced liver disease, especially with higher fibrosis stages (pooled HR 2.50, 95% CI 1.68–3.72) (2, 14, 15).

As a growing amount of evidence suggests that MASLD may independently increase the risk of adverse cardiovascular events like acute coronary syndrome, patients with MASLD may require coronary revascularization interventions such as percutaneous coronary intervention (PCI) and coronary artery bypass grafting (CABG). However, a limited number of studies have evaluated the impact of MASLD on the outcomes of these interventions, possibly due to the novelty of the association between MASLD and cardiovascular disease. This systematic review aims to synthesize current evidence on the influence of MASLD on PCI and CABG outcomes, highlighting the interplay between hepatic and cardiovascular health, identifying potential risk modifiers, and exploring clinical implications for patient management. It also seeks to highlight a growing and previously unrecognized risk factor for cardiovascular disease. Understanding these associations is crucial for developing targeted revascularization strategies to improve cardiovascular outcomes in patients with MASLD.

## Methods

**Figure 1:**
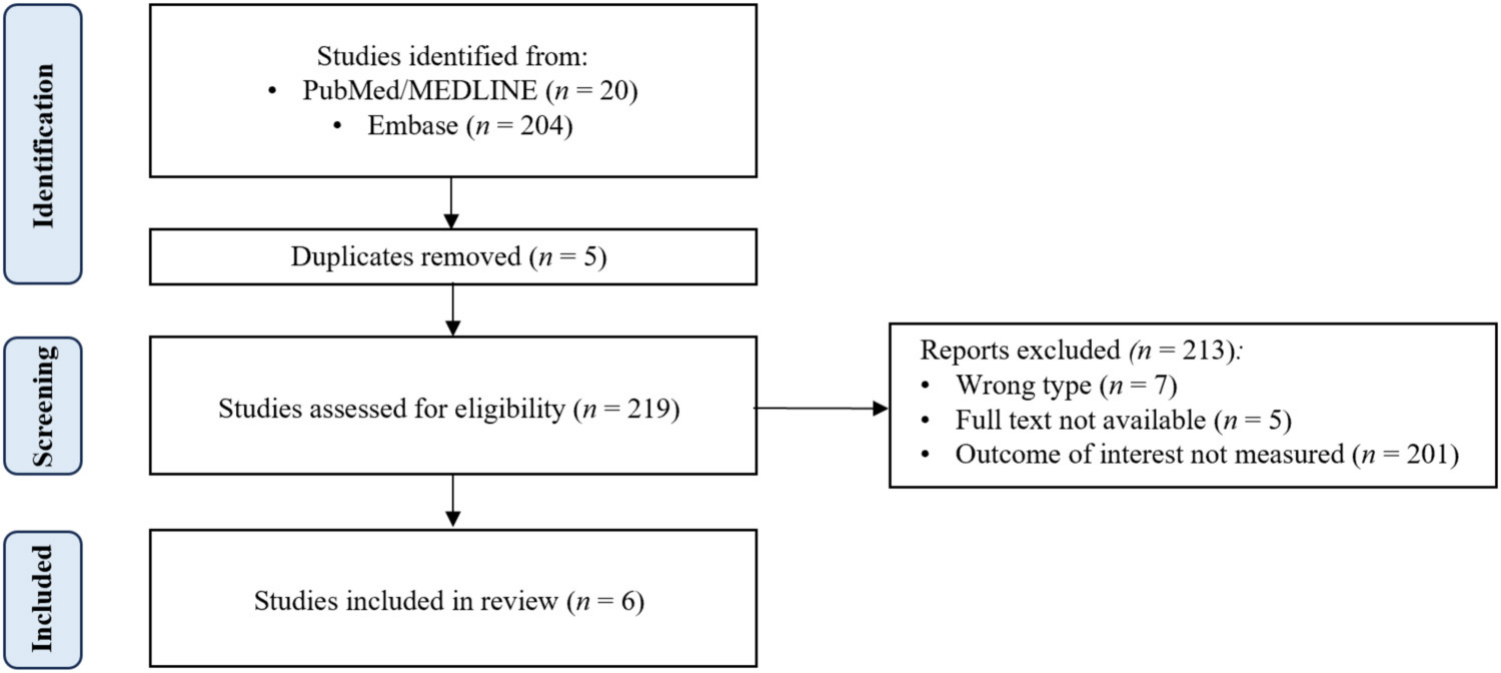
PRISMA diagram.

A comprehensive search of the PubMed/MEDLINE and Embase databases was conducted to identify relevant studies published from August 2015 to August 2025. A combination of Medical Subject Headings (MeSH) terms and text words related to MASLD and cardiovascular revascularization was used. MeSH terms included “metabolic dysfunction-associated steatotic liver disease,” “MASLD,” “non-alcoholic fatty liver disease,” “NAFLD,” “percutaneous coronary intervention,” “PCI,” “coronary artery bypass graft”, “CABG”, “metabolic dysfunction-associated steatohepatitis,” “MASH”, “non-alcoholic steatohepatitis”, and “NASH” (Supplementary Figure S1). Only full-text English-language literature involving human subjects was included, and a range of study types, including prospective cohort studies, experimental studies, population studies, meta-analyses, retrospective cohort studies, clinical trials, and observational studies, were eligible for inclusion. Case reports and series were excluded unless at least five patients were included in their analysis (Figure 1).

The inclusion and exclusion criteria were determined using the “population, exposure, comparators, and outcomes” (PECO) protocol. Studies were included if patients with MASLD who underwent coronary revascularization, either with PCI or CABG, were compared against those without MASLD. Given the novelty of the association between MASLD and CAD and the scarcity of available literature on the topic, studies demonstrating positive, negative, or neutral effects of MASLD on revascularization outcomes were included, with each study's specific outcome of interest included in the overall analysis. Studies were screened by one author and checked by at least one other author. Quality assessments for each study meeting the inclusion criteria were conducted by using the “Newcastle–Ottawa questionnaire for case–control studies” (Supplementary Figure S2).

Data regarding study design, groups, revascularization intervention type, and outcomes were extracted by one author and checked by at least one other. The GRADE approach was used to assess and communicate the overall certainty of evidence (Supplementary Figure S3). After screening, data extraction, and quality assessment, the authors conducted a narrative synthesis of the studies, summarizing the extracted data in a table for easy comparison and review. Studies were grouped based on the type of revascularization performed (i.e., PCI or CABG). The narrative synthesis was then supplemented using additional data available through targeted PubMed/MEDLINE searches.

## Results and discussion

Two hundred nineteen papers from the PubMed/MEDLINE and Embase databases were reviewed. Six met the inclusion criteria (Figure 1). Five studies covered PCI, and one covered CABG. The studies were summarized in Table 1.

**Table 1:** Summary of studies investigating the impact of MASLD on revascularization outcomes, listed alphabetically by author last name.

Author, year	Revasc. method	Study period	Study type	Outcomes measured	Groups	Notable outcomes	Conclusions
Ali et al. 2021 (25)	PCI	2016	NIS cross-sectional	Mortality, LOS, hospitalization cost, MACE	*n* = 2,560 MASLD patients vs. *n* = 427,295 non-MASLD patients	No significant difference in mortality (1.8% vs. 2.2%, *p* = 0.757) or MACE (2.7% vs. 2.9%, *p* = 0.69). MASLD patients were more likely to be younger at admission (61.0 vs. 64.9 years, *p* < 0.001), have a longer LOS (4.65 vs. 3.71 days, *p* = 0.015), and have higher hospitalization costs (115,925 vs. 103,999 USD, *p* = 0.006), compared with non-MASLD patients	MASLD does not affect the rate of in-hospital mortality or MACE in patients undergoing PCI
Emre et al. 2015 (23)	PCI	2012	Prospective cohort	Myocardial perfusion via measuring MBG and STR analysis, MACE	*n* = 111 non-diabetic, STEMI patients with an FLD severity score <3 vs. *n* = 75 non-diabetic, STEMI patients with an FLD severity score ≥3	No difference in TIMI flow grade between the two groups (89% vs. 83%, *p* = 0.201). Patients with FLD score ≥3 were more likely to have absent myocardial perfusion (MBG 0/1, 37% vs. 12%, *p* < 0.0001), absent STR (27% vs. 9%, *p* = 0.001), and a higher in-hospital MACE rate (31% vs. 8%, *p* < 0.0001). By multivariate analysis, FLD ≥3 score was found to be an independent predictor of absent MBG 0/1 (OR 2.856, 95% CI 1.214–6.225, *p* = 0.033), absent STR (OR 2.862, 95% CI 1.242–6.342, *p* = 0.031), and in-hospital MACE (OR 2.454, 95% CI 1.072–4.872, *p* = 0.048)	Despite high rates of TIMI 3 after primary PCI, patients with higher degrees of FLD are more likely to have impaired myocardial perfusion, which may contribute to adverse in-hospital outcomes
Keskin et al. 2017 (18)	PCI	2008–2013	Prospective observational	In-hospital mortality, 3-year mortality	*n* = 169 STEMI patients with no MASLD vs. *n* = 84 STEMI patients with grade 1 MASLD vs. *n* = 71 STEMI patients with grade 2 MASLD vs. *n* = 36 STEMI patients with grade 3 MASLD	In-hospital mortality for grade 0, 1, 2, and 3 MASLD was 4.7%, 8.3%, 11.3%, and 33.9%, respectively. The 3-year mortality rates for grade 0, 1, 2, and 3 MASLD were 5.6%, 7.8%, 9.5%, and 33.3%, respectively. In the multivariable hierarchical logistic regression analysis, in-hospital mortality risks were higher for patients with grade 3 MASLD (OR 4.2, CI 2.0–8.9). In a multivariable Cox proportional regression analysis, the mortality risk was higher for patients with grade 3 MASLD (HR 4.0, CI 3.0–8.1)	Higher degrees of MASLD are associated with higher rates of in-hospital mortality and 3-year mortality
Liu et al. 2021 (24)	PCI	2011–2016	Prospective observational	MACE	*n* = 4003 patients with stable CAD undergoing PCI	During an average follow-up of 5.0 ± 1.6 years, 315 (7.87%) MACE were recorded. Subjects who developed cardiovascular events were more likely to have intermediate or high LFSs, including NFS, fibrosis-4 score, body mass index, AST/ALT ratio, diabetes mellitus score, and AST/ALT ratio. Furthermore, compared with subjects with low scores, those with intermediate plus high score levels had a significantly increased risk of cardiovascular events (adjusted HR ranging 1.57–1.92)	Higher degrees of MASLD are associated with higher rates of MACE
Wong et al. 2016 (19)	PCI	2015	Prospective cohort	Number of coronary artery involvement, mortality, composite cardiovascular outcomes (cardiovascular deaths, non-fatal myocardial infarction, heart failure, or secondary interventions)	*n* = 356 MASLD patients vs. *n* = 256 non-MASLD patients	MASLD patients were more likely to have >50% stenosis in one or more coronary arteries (84.6% vs. 64.1%, *p* < 0.001) and therefore require PCI (68.3% vs. 43.4%, *p* < 0.001). During 3,679 patient-years of follow-up, 47 (13.2%) MASLD patients and 59 (23.0%) patients without MASLD died (age- and sex-adjusted HR 0.36 (95% CI 0.18–0.70, *p* = 0.003). Composite cardiovascular outcomes were similar between groups (36.5% vs. 37.1%; adjusted HR, 0.90 (95% CI 0.69–1.18). Older age and diabetes were the only independent factors associated with cardiovascular events. Only two patients, both in the MASLD group, died of primary liver cancer. No other patients developed liver-related complications	Patients with MASLD are more likely to have multivessel CAD but are not at a higher risk for mortality or adverse cardiovascular outcomes following PCI
Wang et al. 2017 (20)	CABG	2013–2015	Prospective cohort	Changes in inflammatory markers (hsCRP, sCD40l, ICAM-1, MMP-9) and adverse cardiovascular events (mortality, angina pectoris, myocardial infarction)	*n* = 31 MASLD patients vs. *n* = 37 non-MASLD patients	No statistically significant differences in the postoperative inflammatory markers within 24 h of CABG between those with and without MASLD (*p* > 0.05). The expression levels of MMP-9 in MASLD patients at 1 month after operation were significantly higher than those in the non-MASLD group at the same time (*p* < 0.01). Logistic regression analysis revealed that the expression level of MMP-9 was a significant influencing factor for cardiovascular events after CABG (OR 1.182, *p* < 0.05). There were no statistically significant differences between the rates of mortality (3.2% vs. 0%, *p* = 0.356), angina pectoris (9.7% vs. 8.1%, *p* = 0.187), and myocardial infarction (6.5% vs. 5.4%, *p* = 0.276) between those with and without MASLD. However, those with MASLD had a higher total number of adverse cardiovascular events compared with those without (19.4% vs. 13.5%, *p* = 0.29)	MASLD is associated with higher MMP-9 activity levels following CABG, which may correlate with a higher total number of adverse cardiovascular events

ALT, alanine aminotransferase; AST, aspartate aminotransferase; CABG, coronary artery bypass grafting; CAD, coronary artery disease; CI, confidence interval; FLD, fatty liver disease; HR, hazard ratio; hsCRP, high-sensitivity C-reactive protein; ICAM-1, intercellular adhesion molecule-1; LFS, liver fibrosis score; LOS, length of stay; MACE, major adverse cardiac events; MASLD, metabolic dysfunction-associated steatotic liver disease; MBG, myocardial blush grade; MMP-9, matrix metalloproteinase-9; *N*, sample size; NFS, non-alcoholic fatty liver disease score; NIS, National Inpatient Sample; OR, odds ratio; PCI, percutaneous coronary intervention; sCD40l, soluble CD40 ligand; STEMI, ST-segment elevated myocardial infarction; STR, ST-segment resolution; TIMI, thrombolysis in myocardial infarction.

### Impact of MASLD on coronary revascularization outcomes

MASLD is a rapidly growing pandemic that is expected to affect 40% of the adult population worldwide by 2050 (16). It has been associated with a 2–3 times increased risk of cardiovascular disease mortality, irrespective of other common cardiovascular risk factors including hypertension, dyslipidemia, type 2 diabetes, and gender (2). Studies have suggested that MASLD independently increases the risk of developing critical coronary stenosis and coronary plaques, thus necessitating coronary interventions such as PCI or CABG (17). Although evidence suggests MASLD increases the risk of adverse cardiovascular events such as acute coronary syndrome and ischemic strokes (2, 8–15), not many studies have evaluated the impact of MASLD on adverse PCI outcomes. Because MASLD has been shown to increase the risk of adverse cardiovascular events and is correlated to the severity of coronary artery disease, it is not unexpected that patients living with MASLD have poorer outcomes.

Our systematic review yielded a total of five studies that have investigated the impact of MASLD on PCI in-hospital outcomes and one on CABG outcomes since 2015. One study found MASLD to be independently associated with higher rates of in-hospital mortality following PCI. A 2012 prospective observational study by Keskin et al. (18) found the severity of MASLD (grades 0 through 3) to have increasing rates of in-hospital mortality following PCI (grade 0, 1, 2, and 3 MASLD were 4.7%, 8.3%, 11.3%, and 33.9%, respectively). The authors also concluded that patients with grade 3 MASLD were four times more likely to die in the hospital after PCI compared with those without MASLD (HR 4.0, 95% CI 3.0–8.1). Conversely, a retrospective National Inpatient Sample cross-sectional study by Ali et al. (1.8% vs. 2.2%, *p* = 0.757) found that comorbid MASLD to not affect in-hospital mortality compared with those without MASLD (4), and a prospective cohort study by Wong et al. (19) even found those with MASLD to have lower rates of mortality (HR 0.53, 95% CI 0.36–0.78, *p* = 0.001) at 6-year follow-up after discharge. One prospective study by Wang et al. investigated mortality rates following CABG and found no difference in mortality rates between those with and without MASLD (3.2% vs. 0%, *p* = 0.356) (20).

Additionally, conference abstracts have explored PCI mortality outcomes in patients with MASDLD. An NIS analysis abstract by Aboursheid et al. (21) compared the clinical characteristics and outcomes of patients with MASLD undergoing PCI and found MASLD to be independently associated with an increased risk of in-hospital mortality compared with those without MASLD [11% vs. 2.2%, OR 4.9 (95% CI 4.7–5.2), *p* < 0.001], after controlling for age, sex, race, Elixhauser comorbidity index, hypertension, chronic obstructive pulmonary disease, chronic kidney disease (CKD), and history of stroke. Another NIS analysis by Khokhlov et al. (22) also supports the association between MASLD and increased in-hospital mortality [8.9% vs. 1.9%, OR 4.91 (95% CI 4.63–5.21), *p* < 0.001] compared with those without MASLD after comparing for age, sex, race, Charlson comorbidity index, obesity, atrial fibrillation, dyslipidemia, hypertension, peripheral vascular disease, heart failure, CKD, and history of stroke.

Four of our included studies evaluated the effects of MASLD on major adverse cardiovascular events (MACE) following coronary revascularization. A prospective cohort study by Emre et al. (23) found that higher degrees of comorbid MASLD, especially fatty liver disease (FLD) scores ≥3, increase the risk of in-hospital MACE following PCI compared with those without MASLD (31% vs. 8%, *p* < 0.0001). Furthermore, FLD scores ≥3 were more likely to have absent myocardial perfusion (myocardial blush grade 0/1, 37% vs. 12%, *p* < 0.0001) and absent ST-segment resolution following PCI (27% vs. 9%, *p* = 0.001). Another prospective observational study by Liu et al. found that higher degrees of MASLD were associated with higher rates of long-term MACE following PCI, with an average follow-up of 5.0 ± 1.6 years, compared with lesser degrees of MASLD or no MASLD (24). Conversely, the NIS analysis by Ali et al. (25) demonstrated no difference in MACE rate following PCI between those with MASLD and those without (2.7% vs. 2.9%, *p* = 0.69). Wang et al. also found no statistically significant difference in MACE between those with and without MASLD following CABG (19.4% vs. 13.5%, *p* = 0.29) (20).

While the studies identified by our literature search did not address other adverse outcomes related to coronary revascularizations, such as cardiogenic shock, cardiac arrest, in-stent thrombosis, gastrointestinal bleeding, or invasive mechanical ventilation, studies published as conference abstracts have investigated these clinical questions. The unadjusted findings from the NIS analysis abstract by Aboursheid et al. (21) suggest that MASLD is associated with higher percentages of in-hospital cardiogenic shock [7.1% vs. 0.9%, OR 5.1 (95% CI 4.8–5.4), *p* < 0.001], cardiac arrest [4% vs. 0.8%, OR 3.5 (95% CI 3.2–3.8), *p* < 0.001], in-stent thrombosis [0.26% vs. 0.20%, OR 1.3 (95% CI 1.05–1.8), *p* = 0.02], gastrointestinal bleeding [10.7% vs. 5%, OR 2.2 (95% CI 2.1–2.3), *p* < 0.001], and invasive mechanical ventilation [9.5% vs. 2.3%, OR 3.2 (95% CI 3.0–3.4), *p* < 0.001] following PCI, compared with those without MASLD. The NIS analysis abstract by Khokhlov et al. (22) supports the association between MASLD and higher percentages of in-hospital cardiogenic shock [5.4% vs. 0.9%, OR 5.61 (95% CI 5.21–6.05), *p* < 0.001], cardiac arrest [3.6% vs. 0.8%, OR 4.08 (95% CI 3.74–4.45), *p* < 0.001], gastrointestinal bleeding [10.3% vs. 4.5%, OR 2.41 (95% CI 2.29–2.54), *p* < 0.001], and invasive mechanical ventilation [7.8% vs. 2.3%, OR 3.33 (95% CI 3.13–3.54), *p* < 0.001] following PCI, compared with those without MASLD. Finally, a nested case–control study by Lee et al. (26) found that higher fatty liver disease fibrosis scores (NFS), specifically >0.67, were more prevalent in patients with left ventricle dysfunction (left ventricular ejection fraction <40% at PCI) compared with those without (81.0% vs. 33.6%, *p* < 0.001). Baseline higher NFS was significantly associated with LV dysfunction (adjusted OR 1.86, 95% CI 1.36–2.55, *p* < 0.001), and baseline higher NFS and persistent higher NFS at 1 year after PCI were independent predictor of a 5-year CV death, after adjustment for LVEF (adjusted HR 1.42, 95% CI 1.03–1.95, *p* = 0.023; adjusted HR 1.52, 95% CI 1.08–2.64, *p* = 0.033).

### Proposed mechanisms for adverse outcomes following coronary revascularization

MASLD and CAD, though traditionally seen as separate conditions, share core pathophysiological mechanisms rooted in systemic metabolic dysregulation, including insulin resistance, lipotoxicity, chronic inflammation, and atherogenic dyslipidemia. Insulin resistance plays a central role by increasing free fatty acid mobilization and impairing fatty acid oxidation, leading to lipid accumulation in skeletal muscle and liver, which exacerbates hepatic insulin resistance and promotes *de novo* lipogenesis. This cascade leads to the accumulation of toxic lipid species, mitochondrial dysfunction, oxidative stress, and inflammation, ultimately resulting in hepatocyte injury, fibrosis, and organ failure. Adipocyte dysfunction further contributes by flooding multiple organs with fatty acids and fostering a proinflammatory environment marked by elevated cytokines and altered adipokine levels. These processes impair insulin signaling in skeletal muscle, reduce glucose uptake, and perpetuate hyperglycemia and hepatic lipid overload. Chronic hepatic lipogenesis, driven by excess substrate availability and mitochondrial citrate accumulation, contributes to both MASLD and CAD, with key enzymes in this pathway emerging as therapeutic targets. The extent of inflammation, as reflected in markers such as the neutrophil-to-lymphocyte ratio and mTOR pathway activation, correlates directly with CAD severity, underscoring the intertwined nature of metabolic, hepatic, and cardiovascular dysfunction (2).

Although the direct mechanisms linking MASLD to adverse cardiovascular outcomes after coronary revascularization remain unclear, elevated systemic inflammation following the procedure has been associated with worse outcomes. In a single-center, retrospective observational study of nearly 2,000 patients with ischemic heart failure, elevated Systemic Inflammation Response Index, calculated from monocyte, neutrophil, and lymphocyte counts and recognized as a novel marker of chronic low-grade inflammation, was identified as an independent risk factor for major adverse cardiovascular events (MACE) (27). Another single-center, retrospective study involving approximately 500 post-PCI patients also found that the systemic immune-inflammation index, a different surrogate inflammatory biomarker calculated using platelet, neutrophil, and lymphocyte levels, was independently associated with an increased risk of in-stent restenosis (28). Furthermore, a large retrospective study involving over 22,000 consecutive patients with elevated high-sensitivity C-reactive protein who undergo complex PCI found a significantly higher risk of MACE at 1 year following PCI (29). With extrapolations of this data, it is not unreasonable to suspect that the low level of chronic systemic inflammation imposed by MASLD may hinder tissue recovery and routine healing following revascularization. More studies are needed to correlate and potentially identify direct and indirect mechanisms of elevated systemic inflammation following PCI in patients with MASLD.

### Expert recommendations for MASLD patients needing coronary revascularization

The scarcity of studies investigating and comparing coronary revascularization methods, such as PCI and CABG, in patients with MASLD severely hinders claims of superiority or non-inferiority. Previous studies have suggested that MASLD increases the risk of adverse cardiovascular events, and our studies indicate that it may even affect outcomes associated with PCI or CABG, so that risks may be unavoidable with either intervention. Our one included study investigating the mortality associated with CABG found no difference between patients with MASLD and those without MASLD, whereas studies on PCI yielded mixed results. Direct comparisons of PCI and CABG are necessary to determine the preferred intervention methods for individuals with MASLD. Until such studies can be performed, the American Heart Association guidelines recommend that CABG should be preferred over PCI in patients with diabetes and multivessel coronary artery disease involving the left anterior descending artery, who are appropriate surgical candidates, to reduce mortality and the need for repeat revascularizations (30).

Traditional dual antiplatelet therapy (DAPT), which comprises aspirin and a P2Y12 inhibitor, following coronary stenting, largely depends on the type of stent deployed. Bare metal stents should be treated with at least 1 month of DAPT with consideration for continuation longer than 1 month in the absence of high risk or overt bleeding. Drug-eluting stents should be treated with at least 6–12 months of DAPT, depending on bleeding risk or bleeding symptoms. The P2Y12 component of DAPT should be discontinued after 3 months in those with a high risk of bleeding or overt bleeding during DAPT. In patients undergoing CABG, aspirin should be initiated within 6  h postoperatively and continued indefinitely to reduce the risk of saphenous vein graft failure. Select patients may receive DAPT for up to 1 year to improve graft vein patency (30).

Optimal medical management following revascularization has also not been well studied in patients with MASLD. However, clinical recommendations can be extrapolated from studies investigating their safety and efficacy in both MASLD and CAD. Both aspirin and P2Y12 inhibitors are usually safe in patients with MASLD. Aspirin may even ameliorate MASLD and atherosclerosis by inhibiting lipid biosynthesis and elevating catabolic metabolism through the activation of the PPAR*δ*–AMPK–PGC-1*α* pathway. Regular aspirin use has also been suggested to lower the prevalence and progression of fibrosis in patients with MASLD (2). P2Y12 inhibition may also decrease the activation, accumulation, and adhesion of platelets to the liver endothelium, thereby reducing immune cell recruitment to the liver and protecting against liver damage and the development of fibrosis (31). Unfortunately, the optimal duration of antiplatelet therapy has not been extensively studied; however, it is theoretically expected to mirror the guidelines, given the demonstrated safety and efficacy of these drugs.

Ticagrelor is often the preferred oral P2Y12 inhibitor due to a superior reduced rate of death from vascular causes, myocardial infarction, or stroke without an increased risk of overall major bleeding (32); however, the choice of P2Y12 inhibitor in chronic liver disease, including MASLD, is less well-defined. An *in vitro* study found the potency of aspirin, cangrelor, and ticagrelor in patients with MASLD cirrhosis to be similar to that of healthy controls (33). A murine model suggested that ticagrelor, not clopidogrel, can attenuate hepatic steatosis and reduce the non-alcoholic fatty liver disease activity score in mice with MASLD by decreasing hepatic lipogenesis and endoplasmic reticulum stress markers, as well as inflammation-related genes (34). However, another murine model suggested that clopidogrel could mitigate hepatic steatosis and downregulate the expression of lipogenic, profibrotic, and proinflammatory genes, while enhancing the phosphorylation of adenosine monophosphate-activated protein kinase and acetyl-coenzyme A carboxylase (35). Overall, P2Y12 inhibitors appear to be effective in treating patients with MASLD. More evidence regarding the extracardiac and hepatic benefits is needed to determine the best choice for patients living with MASLD.

Beta-blockers are a core component of post-acute coronary syndrome management. They are generally considered safe in patients with chronic liver disease, as evidenced by non-selective beta-blocker prescription for ascites control in patients with decompensated cirrhosis (36). Some beta-blockers, particularly atenolol and metoprolol, should be avoided in patients with metabolic disorders due to their potential to cause weight gain through decreased energy expenditure. Beta-blockers with additional alpha-receptor antagonism, such as carvedilol and labetalol, may cause less weight gain and are better suited for patients with MASLD (2, 37). Murine models have yielded mixed results regarding the benefits or harms of beta-blockade related to MASLD. One study suggested that beta-blockade may reduce insulin resistance, improve dyslipidemia, and increase the expression of hepatic GLUT4, whereas another study suggested that it may induce MASH in patients with MASLD (2, 38, 39). Overall, beta-blockers are likely safe for use in patients with MASLD; however, further studies are needed to assess their long-term hepatic and metabolic effects.

Statins have been demonstrated to be safe and effective in treating both coronary artery disease and MASLD (2). According to the American College of Cardiology, all patients undergoing PCI or CABG for acute coronary syndrome or coronary artery disease should be initiated on high-dose statin therapy, if not already prescribed, with a target reduction in low-density lipoprotein cholesterol (LDL-C) of at least 50% compared with their baseline level. In very high-risk patients with a history of one or more major cardiovascular disease events or one major atherosclerotic cardiovascular disease event and multiple high-risk conditions, reduction of LDL-C to <55 mg/dl should be targeted (40). The European Society of Cardiology recommends that all patients be targeted to an LDL-C reduction to <55 mg/dl and ≥50% vs. baseline (41). If these targets cannot be reached, adjunctive medications such as ezetimibe or PCSK9 inhibitors can be initiated (40).

The latest European Association for the Study of the Liver–European Association for the Study of Diabetes–European Association for the Study of Obesity (EASL–EASD–EASO) guidelines for the management of MASLD recommend the use of statins for cardiovascular indications, rather than specifically for hepatic benefit, as their safety has been consistently demonstrated in patients with liver disease. A limited number of case–control studies have associated statins with a reduced risk of MASLD, MASH, and liver fibrosis, as well as a reduced risk of hepatic decompensation, mortality, and hepatocellular carcinoma in patients with cirrhosis. However, no large randomized controlled trials with histological evidence exist to probe their effectiveness in MASLD or MASH (2, 42).

## Study limitations

While our systematic review makes a significant contribution to hepatological and cardiological literature by highlighting a highly relevant and underexplored clinical topic, it has its limitations. Firstly, the study is constrained by the small number of available and diverse studies published during the search timeframe. We employed broad inclusion criteria to capture any adverse outcomes of coronary revascularization in patients with MASLD from all recent literature. Given the scarcity of information on this topic, it was essential to gather and synthesize as much data as possible. Additionally, non-English-language studies were excluded as the authors are primarily English speaking, and extrapolating translated data imposes additional bias. Second, our systematic review was not registered with the International Prospective Register of Systematic Reviews (PROSPERO) as the literature search and data extraction process had already begun before attempting registration, which may introduce a reporting bias. However, when briefly compared with systematic reviews in the PROSPERO database during attempted submission, no similar studies appeared to exist. Finally, our study is limited by its nature as a systematic review, which introduces the risk of biases, such as selection bias, attrition bias, and selective outcome reporting, despite attempts to mitigate these biases through multi-reviewer article and data checking at multiple stages of the review process.

## Conclusions

MASLD has been associated with increased risk of adverse cardiovascular events and may pose an increased risk of in-hospital and long-term mortality following PCI. Risks for cardiogenic shock, cardiac arrest, in-stent thrombosis, gastrointestinal bleeding, or invasive mechanical ventilation following PCI may also be increased. One study proposed that MASLD does not have a negative impact on post-CABG outcomes; however, more studies are needed to corroborate this finding and determine the optimal coronary revascularization method in patients with MASLD. Further studies should also investigate optimal medical management of patients with MASLD following PCI or CABG to mitigate long-term mortality and adverse event risk in these patients.

## Data Availability

The original contributions presented in the study are included in the article/Supplementary Material, further inquiries can be directed to the corresponding authors.
