# Supplementary material for: Outcomes of coronary revascularization in patients with metabolic dysfunction-associated steatotic liver disease: a systematic review
Source: Front Cardiovasc Med. 2025 Aug 26;12:1609071. doi: 10.3389/fcvm.2025.1609071 (PMC12417393; doi:10.3389/fcvm.2025.1609071)
Supplement: Supplementary file 1 [file Datasheet1.pdf]

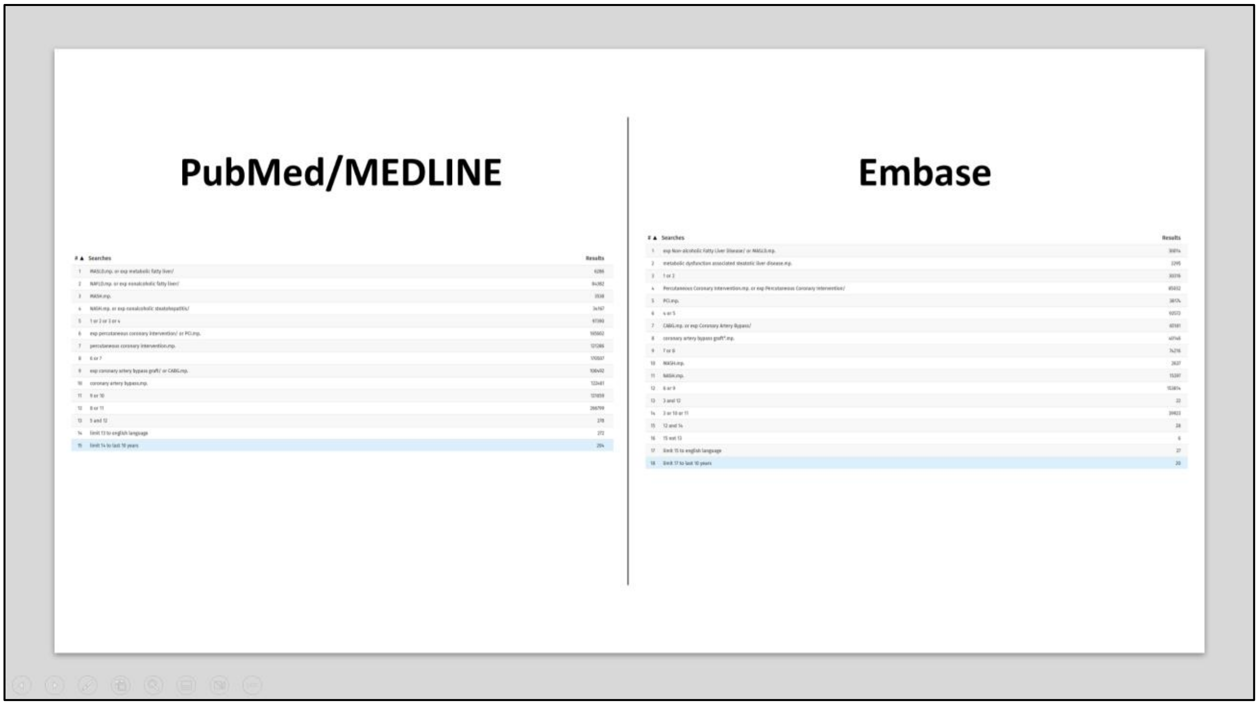

PubMed/MEDLINE

#	Searches	Results
1	MASLD.mp. or exp metabolic fatty liver/	6286
2	NAFLD.mp. or exp nonalcoholic fatty liver/	94382
3	MASH.mp.	3538
4	NASH.mp. or exp nonalcoholic steatohepatitis/	34167
5	1 or 2 or 3 or 4	97390
6	exp percutaneous coronary intervention/ or PCI.mp.	165652
7	percutaneous coronary intervention.mp.	121286
8	6 or 7	170507
9	exp coronary artery bypass graft/ or CABG.mp.	106492
10	coronary artery bypass.mp.	122461
11	9 or 10	121858
12	8 or 11	266799
13	5 and 12	278
14	limit 13 to english language	272
15	limit 14 to last 10 years	254

Embase

#	Searches	Results
1	exp Non-alcoholic Fatty Liver Disease/ or MASLD.mp.	30814
2	metabolic dysfunction associated steatotic liver disease.mp.	3295
3	1 or 2	30316
4	Percutaneous Coronary Intervention.mp. or exp Percutaneous Coronary Intervention/	85032
5	PCI.mp.	38174
6	4 or 5	93573
7	CABG.mp. or exp Coronary Artery Bypass/	67181
8	coronary artery bypass graft*.mp.	40746
9	7 or 8	74216
10	MASH.mp.	2637
11	NASH.mp.	15397
12	6 or 9	153814
13	3 and 12	22
14	3 or 10 or 11	39823
15	12 and 14	28
16	15 not 13	6
17	limit 15 to english language	27
18	limit 17 to last 10 years	20

Supplemental Figure 1. Full search strategy.

	Studies (year)					
**Newcastle-Ottawa Quality Assessment Criteria**	Ali et al. (2021)	Emre et al. (2015)	Keskin et al. (2017)	Liu et al. (2021)	Wong et al. (2016)	Wang et al. (2017)
A. Selection (maximum of four stars)						
1. Is the case definition accurate?	★	★	★	★	★	★
2. Representativeness of the cases	★	★	★	★	★	★
3. Selection of controls	★	☆	★	★	★	★
4. Definition of controls	★	★	★	★	★	★
B. Comparability (maximum of two stars)						
1. Comparability of cases and controls based on the design or analysis	★★	★★	★★	★★	★★	★☆
C. Outcome (maximum of three stars)						
1. Ascertainment of exposure	★	★	★	★	★	★
2. Same method of ascertainment for cases and controls	★	★	★	★	★	★
3. Non-response rate	★	☆	☆	☆	★	☆
Total (maximum of nine stars)	9	7	8	8	9	7

Supplemental Figure 2. Individual risk of bias assessments for each included study.

Impact of MASLD on PCI and CABG Outcomes

Certainty assessment						
**Participants (studies) Follow-up**	**Risk of bias**	**Inconsistency**	**Indirectness**	**Imprecision**	**Other Considerations**	**Overall Certainty of Evidence**
(5 non-randomised studies)	Not serious	Very serious[a]	Not serious	Not serious	Strong association	⨁⨁⨁◯ Moderate[a]

Explanations

a. While four of the six studies found the presence of MASLD to be associated with more adverse outcomes following coronary revascularization compared to without, two studies found the presence of MASLD to have no effect. Furthermore, each of the studies demonstrated this using different measured outcomes, i.e., in-hospital mortality, 3-year mortality, higher MMP-9 activity, coronary reperfusion rates, etc.

Supplemental Figure 3. Overall certainty of evidence assessment using the GRADE approach. The assessment was performed using the GRADEpro GDT online tool.
